# Two Decades of Nanotoxicology: Evolution, Impact, and Future Challenges

**DOI:** 10.1021/acsomega.6c04845

**Published:** 2026-08-21

**Authors:** Marília Magalhães Gonçalves, Igor José Boggione Santos, Taíla Veloso de Oliveira, Ana Paula Madureira, Kely de Paula Correa, Marcos Silva de Sousa, Bruna Mara Aparecida de Carvalho Mesquita, Jane Sélia dos Reis Coimbra

**Affiliations:** † Departamento de Tecnologia de Alimentos, 28120 Universidade Federal de Viçosa, Viçosa 36570-900, Brazil; ‡ Departamento de Química, Biotecnologia e Engenharia de Bioprocessos, 74383 Universidade Federal de São João del-Rei, Ouro Branco 36420-000, Brazil; § Departamento de Engenharia de Biossistemas, Universidade Federal de São João del-Rei, São João del-Rei 36301-160, Brazil; ∥ 420698 Empresa de Pesquisa Agropecuária de Minas Gerais, EPAMIG, Belo Horizonte 36045-560, Brazil; ⊥ Departamento de Ciências Naturais, Universidade Federal de São João del-Rei, São João del-Rei 36301-160, Brazil; # Institute of Agricultural Sciences, Universidade Federal de Minas Gerais, Montes Claros 39.404-547, Brazil

## Abstract

The rapid expansion of nanotechnology, with over 11,100 products and 100,000 patents, has raised safety concerns for organisms and ecosystems. Nanotoxicology emerged from ultrafine particle studies in the 1990s and was formally established in 2004. This review synthesizes approximately 160 publications and regulatory documents, selected from 220 compiled, to analyze the field’s evolution from 2004 to 2025. Early studies focused on nanostructures in medicine and their effects on organs and tissues, driving research aimed at mitigating risks. Current studies increasingly address the molecular mechanisms underlying nanostructure toxicity, which arise from dynamic interactions among physicochemical properties, exposure conditions, biological transformations, and environmental fate. Research extends beyond human health to assess long-term environmental impacts. Artificial intelligence and machine learning have enabled predictive models linking physicochemical properties to toxicological outcomes, although reliability remains limited by data set heterogeneity, restricted external validation, and overfitting. Bibliometric analysis revealed a publication peak around 2014–2016 (>380 articles/year), followed by a decline through 2023–2024 and partial recovery in 2025, reflecting diversification into nanosafety, safety-by-design, nanoecotoxicology, and AI-based prediction. Standardized protocols, harmonized data sets, and globally accepted safe exposure limits remain unavailable. Future progress depends on integrating biologically and environmentally relevant models, robust computational approaches, and harmonized regulatory frameworks to advance sustainable nanotechnology.

## Graphical abstract

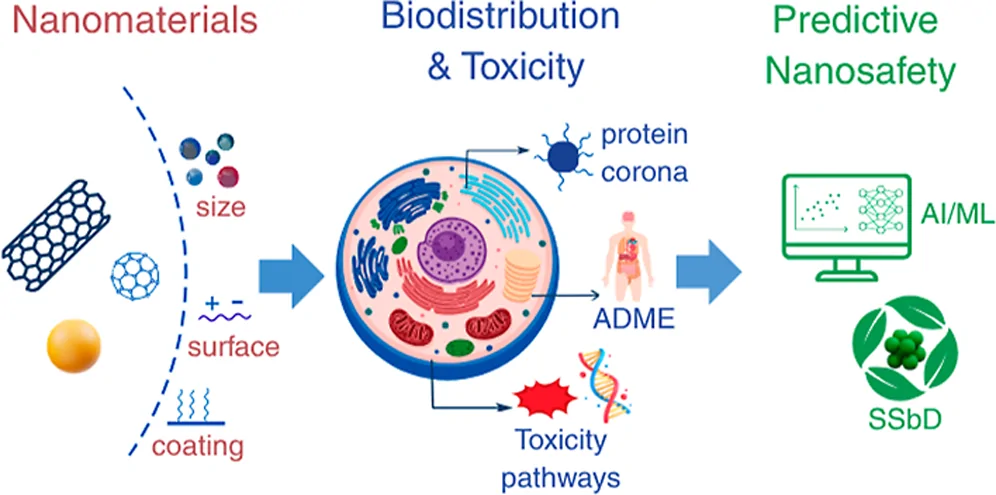

## Introduction

In recent years, from 2000 to 2025, the rapid expansion of nanotechnology has led to the development and application of nanomaterials across sectors such as medicine, electronics, food, agriculture, and cosmetics.^1–3^ Nanotechnology is considered a disruptive technology, as its concepts and applications impact the environment, human life, and various sectors of industry, particularly across the product life cycle, business, and market. Compared with materials with equivalent compositions in terms of micrometric or macroscopic dimensions, nanomaterials have distinct physicochemical and/or biological properties owing to their high surface-to-volume ratio and quantum effects.^4^ However, the increasing use of these materials and their nanocomposites in various consumer products has led to potential human exposure to these nanostructures through inhalation, ingestion, and skin contact. This exposure has been associated with adverse side effects attributed to nanotoxicity, particularly in systems such as the central nervous, circulatory, gastrointestinal, and respiratory systems.^5,6^

Nanotoxicology emerged in the 1990s when toxicologists expanded their research on the pulmonary effects of airborne particles to investigate the influence of engineered nanomaterials, such as metal oxides and carbon nanotubes.^7^ Subsequently, nanotoxicology was supported by European legislation through the implementation of the REACH regulation (Registration, Evaluation, Authorization, and Restriction of Chemicals), which began requiring systematic assessment of the toxicity of chemical substances, including nanomaterials.^8^ To date, various approaches, including computational methods and in vitro and in vivo assays, have been developed to study nanotoxicity. These strategies enable the evaluation of cytotoxicity, genotoxicity, oxidative stress, and inflammatory responses, thereby contributing to understanding the adverse effects of nanomaterials at various biological levels.^9^

However, despite these advances, nanotoxicology faces practical limitations. The great heterogeneity of nanomaterials, along with variations in composition, size, shape, functionalization, and aggregation of the same compound, makes it challenging to standardize toxicological assays, thereby hindering comparability between studies.^10^ Additionally, many traditional in vitro models fail to accurately replicate the complex biological interactions of living organisms, resulting in a low correlation between in vitro and in vivo studies. Another critical gap concerns the lack of data on chronic effects and bioaccumulation and the limited application of physiologically based models to nanomaterials such as physiologically based pharmacokinetic models (PBPKs).^11,12^

The available knowledge remains quite fragmented across distinct research areas, including physicochemical characterization, biological interactions, toxicological mechanisms, environmental impacts, regulatory evaluation, and predictive modeling.^9^ Therefore, researchers developing new nanomaterials often face difficulties in identifying which toxicological parameters to prioritize, interpreting experimental results across different studies, and determining whether existing testing strategies are sufficient to ensure safety from production onward.^13^ As a result, an integrative perspective that connects these developments, identifies persistent challenges, and examines how the field has evolved toward more comprehensive and predictive assessment strategies remains necessary. Although several reviews have addressed specific aspects of nanotoxicology, few have integrated its historical evolution with advances in exposure characterization, mechanistic toxicology, environmental risk, regulatory science, and predictive computational approaches.

Thus, this review aims to provide an overview of the evolution of nanotoxicology over the past two decades (2004–2025), tracing its conceptual development, methodological advances, and current challenges. This work integrates important milestones, such as human and environmental exposure to nanostructures, advances in understanding the molecular and cellular mechanisms of nanotoxicity, environmental risk assessment, regulatory developments, and the growing use of computational approaches.^14^ It also discusses how emerging artificial intelligence and machine learning (AI/ML) tools contribute to predictive toxicology and proposes a framework for sustainable toxicological testing that considers the scientific and regulatory perspectives. By bringing these developments together, this review seeks to provide a broader understanding of the current landscape and future directions of nanotoxicology.

### Literature Search

A narrative approach, rather than a systematic approach, was adopted for this review because of the breadth and interdisciplinary nature of nanotoxicology research over the two decades under review, encompassing multiple exposure pathways, biological systems, and methodological frameworks. This procedure enabled a more critical synthesis that integrated conceptual advances, methodological developments, and emerging challenges across various areas of nanotoxicology. Therefore, the selection of articles prioritized thematic relevance, methodological contribution, and historical significance, with the aim to provide an overview of the historical evolution of the field.

The literature survey was conducted primarily through the CAPES Journal Portal (2024),^15^ which provides access to major international scientific databases, such as Web of Science, Scopus, and ScienceDirect. Additional publications were identified through Google Scholar^16^ searches and analysis of the reference lists of key articles. To ensure the inclusion of recent advances in the field, targeted supplementary searches were performed during manuscript preparation whenever additional evidence was needed to elaborate on specific topics within the scope of the review.

The search focused on publications supporting the discussion of scientific advances that shaped the evolution of nanotoxicology between 2004 and 2025, including exposure pathways, biodistribution, absorption, distribution, metabolism, and excretion (ADME) processes, toxicity mechanisms, protein corona formation, environmental fate, ecotoxicology, regulatory developments, and the application of artificial intelligence (AI) and machine learning (ML) in nanotoxicology. Searches employed combinations of the keywords: “nanotoxicology”, “nanosafety”, “nanomaterial toxicity”, “biodistribution”, “ADME”, “protein corona”, “environmental fate”, “ecotoxicology”, “risk assessment”, “regulation”, “nanoinformatics”, “artificial intelligence”, and “machine learning” combined with Boolean operators (AND/OR) when appropriate.

Publications identified through database searches and reference analysis underwent an initial assessment on the basis of their titles, abstracts, and alignment with the objectives of this review. Original research articles, review papers, and regulatory documents considered potentially relevant were subsequently organized using reference management software Mendeley. The authors compiled 220 scientific publications and regulatory documents during the writing of this narrative review, following the search strategy and selection criteria described above. Of these, 161 were selected and cited in the final paper according to their contribution to the objectives and scope of this work. Supplementary searches followed the same selection criteria to ensure consistency throughout the literature selection process. These criteria included thematic relevance to these objectives, scientific contributions to the development of nanotoxicology, and historical importance within the field. Duplicate records, publications not directly related to nanotoxicology, and studies that did not contribute to an understanding of the historical evolution of the field or current advances were excluded.

The selected literature underwent qualitative analysis and was organized in terms of both the chronological order of contributions to the evolution of nanotoxicology and their relationships with the main topics addressed in this review. The categories included exposure assessment, biodistribution and ADME processes, toxicity mechanisms, protein corona, environmental fate and ecotoxicology, regulatory developments, and artificial intelligence- and machine learning-based predictive approaches. The qualitative synthesis of the selected literature identified key scientific advances, methodological trends, emerging challenges, and shifts in research priorities throughout the evolution of nanotoxicology.

### Exposure to Nanomaterials

Humans and the environment have been exposed to nanostructures since the beginning, as nanostructures can originate from natural sources such as sea salt and volcanic dust. However, contact with these materials has increased significantly over the past century, primarily because of the development of a new generation of anthropogenic nanostructures. These sources can be byproducts or residues in industrial processes, for example, from the burning of fossil fuels or intentionally produced engineered nanostructures.^17–19^

Because of their unique properties, engineered nanomaterials have been developed for specific purposes. Material manipulation at the nanoscale, which considers quantum and surface effects to create functional materials, falls within the scope of nanoscience, and the production of nanomaterials lies within the realm of nanotechnology.^20^

Currently, engineered nanostructures are widely present in the environment because many products containing them are available on the market. StatNano (2026),^21^ a global initiative that provides comprehensive statistics and information on nanotechnology, reports more than 11,100 nanotechnology products available worldwide in 2026. They are distributed across various sectors, including medicine, cosmetics, electronics, textiles, food, agriculture, and sports. A Google Patents search (2026)^22^ for the terms “nanotechnology,” “nanostructures,” “nanomaterials,” or “nanoparticles” returned more than 100,000 patents with these terms in the title through 2026. As the production and marketing of these products continue to expand, human and environmental exposure also increases. The main pathways through which nanomaterials interact with humans include skin penetration, ingestion, and inhalation.^23^ The main pathways of human and environmental exposure to nanostructures, including their release and transfer throughout the nanomaterial life cycle, are summarized in Figure 1.

**Figure 1:**
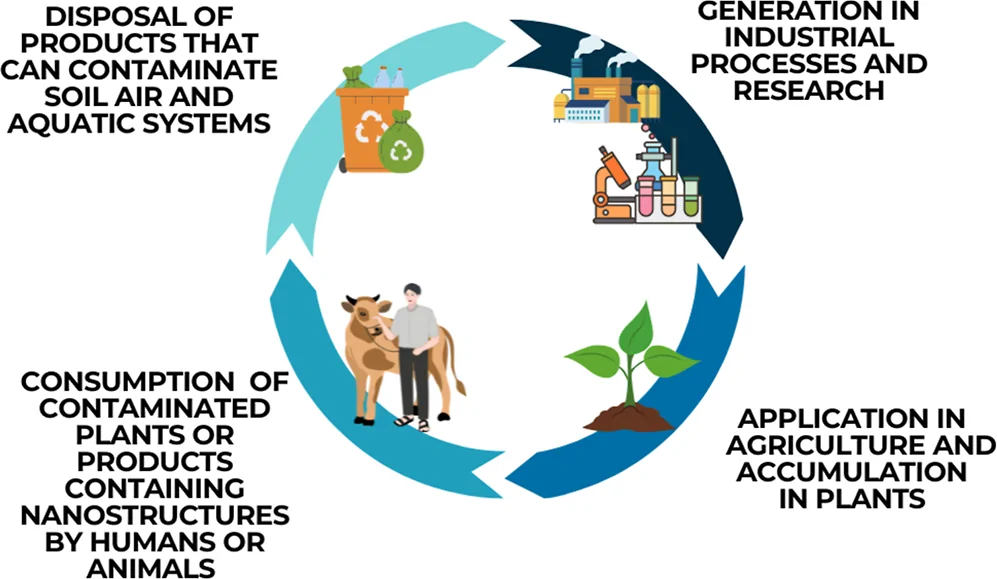
Routes of exposure of humans and the environment to nanostructures.

In these scenarios, occupational exposure is among the most significant routes of human contact with engineered nanomaterials, particularly during manufacturing, handling, packaging, processing, and disposal activities. However, exposure is not determined solely by the presence of nanomaterials in the workplace. Their release can occur throughout the product life cycle and is influenced by factors such as the physical state of the material (powders, suspensions, or solid matrices), process energy, nanomaterial concentration, environmental conditions, and the effectiveness of controls including ventilation and containment systems. Furthermore, nanomaterial exposure is a multistep process involving release, emission into the surrounding environment, transport, and final contact with the workers. High-energy activities, such as synthesis, spraying, and machining, generally result in greater particle emissions than low-energy handling operations, leading to substantial variability across exposure scenarios.^24^

This variability has been demonstrated in workplace exposure studies involving TiO_2_ nanoparticles. Xu et al. (2016)^25^ reported airborne concentrations of approximately 1.0 × 10^5^ particles/cm^3^ in a packaging workshop compared with 1.2 × 10^4^ particles/cm^3^ in a milling workshop. Similarly, Koivisto et al. (2018)^26^ reported that although dip-coating resulted in negligible nanoparticle release, air-blade drying led to measurable worker exposure, with respirable TiO_2_ concentrations of approximately 4.2 μg/m^3^ in the breathing zone.

Exposure is not restricted to the occupational settings. Consumers may also come into contact with engineered nanomaterials throughout the product life cycle, including during product use and disposal. Contact may occur through products, such as cosmetics, food packaging, textiles, and medical devices. However, assessing consumer exposure remains challenging because detailed information regarding nanomaterial content, release rates, and exposure conditions is often limited, particularly for products in liquid or solid form. Consequently, both occupational and consumer exposure scenarios must be considered when evaluating the potential risks associated with the engineered nanomaterials. In contrast to occupational settings, where quantitative exposure measurements have been reported for specific industrial processes, comparable estimates for consumer exposure remain scarce because the nanomaterial content, release rates, and patterns of use are still insufficiently characterized.^27^

Notable exceptions exist for a small number of well-studied materials for which quantitative consumer exposure data are available. Quantitative data are available for oral exposure, particularly regarding titanium dioxide as a food additive, designated E171 under the European Union’s food additive numbering system (equivalent to INS 171 under the Codex Alimentarius international system). A recent systematic review and meta-analysis estimated that the mean dietary TiO_2_ intake ranged from 0.045 to 10.5 mg/kg body weight per day globally, with a lifelong weighted average of approximately 1.43 mg/kg body weight per day; notably, children were significantly more likely to be exposed to TiO_2_ than adults.^28^ Regarding dermal exposure, an in vitro study using human epidermal membranes and a sunscreen formulation containing micronized zinc oxide revealed that less than 0.03% of the applied zinc content penetrated the epidermis after 24 h of exposure.^29^ These findings were subsequently corroborated under more realistic conditions: repeated daily application of sunscreens containing ZnO nanoparticles to human volunteers over several days resulted in neither nanoparticle penetration through the stratum corneum nor any detectable morphological or redox changes.^30^

Furthermore, unlike conventional chemicals, the nominal exposure concentration does not necessarily represent the biologically relevant dose, since toxicological responses are more closely associated with the internal dose that effectively reaches tissues and cells. This internal dose depends on factors such as cellular absorption, biodistribution, and biological interactions. Additionally, there is still no consensus on the most appropriate metric to express exposure and dose to nanomaterials. Although mass concentration is typically used, evidence suggests that particle number and surface area may better reflect dose–response relationships for certain nanomaterials. This lack of standardization hinders comparisons between studies and represents a significant challenge for risk assessment and the establishment of harmonized regulatory frameworks.^31^

As illustrated in Figure 1, exposure to nanomaterials must be considered throughout their entire life cycles. In addition to direct exposure pathways, nanomaterials can be released during production, incorporated into commercial products, accumulate in the environment, and subsequently enter food chains, thus increasing the opportunities for human and ecological exposure. Therefore, nanotoxicological assessments must consider not only direct biological effects but also the environmental fate, persistence, and potential transfer between ecosystems. Despite the difficulties in understanding exposure scenarios, this is a fundamental step in nanotoxicological assessment since the pathway, magnitude, duration, and context can directly influence the biodistribution of nanomaterials, biological interactions, and potential toxicological outcomes. Considering these aspects, it is possible to interpret the heterogeneous results reported in nanotoxicological studies to support reliable risk-assessment strategies.^24^

### First Decade of Nanotoxicological Studies, from 2004 to 2014

As in classical toxicology, understanding the relationships among risk, toxicity, and exposure is essential in nanotoxicological studies. Therefore, to predict and manage the potential risks posed by nanostructures to human and animal health and the environment, determining exposure levels, characterizing nanostructures, and identifying potential toxicity are necessary.^32^

However, while traditional toxicology is strongly driven by the relationship between compound dose and organism response, nanotoxicology adds complexity to this relationship, stemming from physicochemical properties intrinsic to nanostructures such as size, morphology, composition, surface area, state of aggregation/agglomeration, and environmental transformations (such as aging and protein corona formation) that directly modulate their biological interaction, as illustrated in Figure 2. Consequently, nanotoxicological risk assessment requires a multidimensional approach that considers both the exposure levels and the specific properties of nanomaterials.

**Figure 2:**
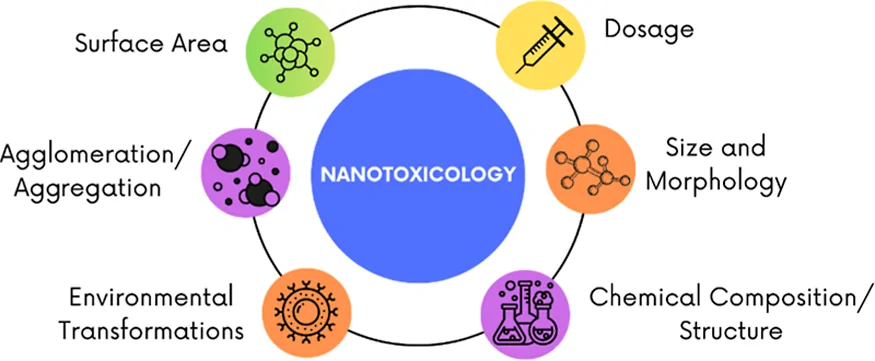
Elements evaluated in nanotoxicology.

Nanotoxicological studies have evolved to understand the factors cited above, reflecting the growing importance of nanotechnology and its applications. Although investigations into the biological effects of ultrafine and nanoscale particles were already conducted during the 1990s, particularly in the context of airborne particulate matter, the term nanotoxicology was formally introduced by Donaldson et al. (2004),^33^ helping to establish the field as a distinct area of research. From that point onward, the number of studies specifically addressing the safety, toxicity, and exposure of engineered nanomaterials has increased substantially. However, toxicological investigations have not kept pace with the development and application of nanotechnology.

In 2019, the number of articles in the PubMed database on the toxicity, safety, or exposure levels of nanostructures was 2.7 times lower than that on their development and applications.^34^ This initial discrepancy, already highlighted in several reviews, reflects the absence of specific regulations and structured protocols for the systematic evaluation of safety.

Several literature reviews highlighted the rapid evolution of nanotechnology and its applications in the first decade, although no specific regulations or protocols have been developed to guarantee the safety of nanocompound applications. In this initial period, three issues emerged as central to guiding the field: (i) the fear of public rejection of nanotechnology in the face of uncertainties about potential risks,^35^ and (ii) the need for collaboration between academia, the regulatory sector, and society to ensure safe and socially acceptable development.^36,37^ As highlighted by Service (2004), the interfacial properties of nanostructures, which make them promising for use in various areas, may be the same features that can contribute to toxic effects on organisms.^35^

In 2005, the first experimental studies on the toxicological effects of nanostructures were published.^37–39^ Some studies^3^ have focused on the impact of nanostructures on the lung, as previous research in occupational environments has shown that fine particles dispersed in the air are associated with lung damage. The development and adaptation of methodologies for characterizing and determining the harmful consequences of nanostructures were subsequently described. Simultaneously, a growing number of investigations have focused on materials with biomedical potential, driven by the need to ensure safety in diagnostic and therapeutic applications.^40–42^

The first nanomaterials analyzed in the nanotoxicological tests were quantum dots, carbon nanotubes, and silica nanoparticles. Toxicological effects were evaluated in vitro using distinct cell types and in vivo in rats and pigs. Later, metallic nanostructures and fullerenes were studied, and new in vivo models, such as zebrafish, carp, and crustacean embryos, were assessed.^43–45^ These studies yielded important results, such as the strong dependence of nanostructures on their size and shape, their ability to accumulate in filtering organs, and their capacity to induce oxidative stress and activate inflammation.

Among these early investigations, studies on quantum dots (Qds) highlighted the importance of surface characteristics in determining biological interactions and toxicological responses. Chang et al. (2006)^46^ evaluated the cytotoxicity of cadmium selenide and cadmium sulfide quantum dots in human cancer cells. The cytotoxicity of Qds was associated with the degree of chemical compound degradation and the number of particles capable of entering cells. Coating nanostructures with the polymer polyethylene glycol (PEG) reduces their cellular uptake, thereby decreasing the toxicity of quantum dots. The Qd nanostructures were 25 nm in size, reaching 37 nm with the coating. Similar findings emerged from biodistribution studies. Fischer et al. (2006)^47^ reported the biodistribution and elimination of semiconductor Qd coated with mercaptoundecanoic acid or bovine serum albumin in rats. The authors observed (1) no excretion of the nanostructure in feces or urine for up to 10 days; (2) the highest concentration of the particles in the liver; and (3) a redistribution mechanism of the nanostructures or their reabsorption at slow rates. In parallel, Ryman-Rasmussen et al. (2006)^48^ studied the ability of commercially available Qd to penetrate the intact skin of pigs. The Qd nanostructures (a) were spherical particles with a diameter of 4.6 nm and ellipsoidal particles measuring 12 nm in length and 6 nm in width; (b) had surfaces modified with neutral (PEG), anionic (carboxylic acid), or cationic (PEG-amine, PEG-NH_2_) coatings; and (c) could overcome the skin barrier, indicating a possible route for human and animal exposure. Before these experiments were performed, researchers believed that the skin route would be possible only if the skin was injured.

Studies involving carbon nanotubes have contributed to a broader understanding of the nanotoxicity mechanisms. Investigations with these nanomaterials have demonstrated that the metallic compounds present can act as catalysts for oxidative stress. Kagan et al. (2006)^49^ determined the ability of iron-rich carbon nanotubes (26 wt % iron) or purified carbon nanotubes with iron (0.23 wt % iron, single-layer carbon nanotubes) to generate oxidative stress in macrophages. The authors reported that (1) the average diameter of the nanotubes ranged from 1 to 4 nm; (2) the redox activity of the iron-rich nanotubes increased, indicating that iron is a catalyst for oxidative stress, thus contributing to cytotoxicity; (3) oxidative stress could be quantified by means of radical intermediates via electron paramagnetic resonance (EPR) spectroscopy; and (4) the intracellular production of superoxide and nitric oxide radicals was measured by flow cytometry.

Other studies have highlighted the importance of nanomaterial composition. Cheng et al. (2007)^44^ reported a delay in zebrafish embryo hatching, which they attributed to the use of copper and nickel residues as catalysts in the production of double-walled carbon nanotubes, as these compounds have been shown to inhibit hatching. In contrast, Singh et al. (2006)^41^ evaluated the toxicity of water-soluble carbon nanotubes and single-walled carbon nanotubes with diameters of 20–30 nm and lengths of 0.5–2 μm, functionalized with the chelating agent diethylenetriaminepentaacetic acid. The functionalized nanotubes were intravenously administered to the rats. The nanostructures were not retained in the organs of the reticuloendothelial system of the animals, and they were eliminated by renal excretion while still intact. These findings reinforce the importance of complete characterization and impurity control in the production of nanostructures.

Beyond the influence of composition and surface characteristics, several studies have demonstrated that dose and particle geometry play important roles in determining the toxicological effects of carbon-based nanomaterials. The toxicity of single-walled carbon nanotubes (SWCNT), multiwalled carbon nanotubes (MWCNT), and fullerene was evaluated in vitro in alveolar macrophages. The dose and particle geometry influence the development of necrosis and cell degeneration.^39^ The persistence of multiwalled carbon nanotubes in the lungs of rats for up to 2 months led to tissue damage and elevated levels of inflammatory cytokines. These findings were later complemented by evidence suggesting that the consequences of carbon nanotube exposure may extend beyond local pulmonary damage.^37^ Li et al. (2007)^50^ verified the effects of single-walled carbon nanotube exposure in the lungs of mice. The animals were exposed to SWCNT at doses of 10–40 μg/mouse via a single intrapharyngeal instillation. Changes in parameters related to cardiovascular diseases, such as the formation of atherosclerotic plaques, were observed after 7, 28, and 60 days, indicating the potential for systemic effects of SWCNT.

Biodistribution studies have reinforced the association between exposure to nanomaterials and systemic effects. Xie et al. (2010)^51^ studied the toxicity and biodistribution of silica nanostructures applied intravenously to rats. Researchers have observed accumulation in organs such as the liver, lungs, and spleen driven by histological changes in the liver and by macrophage endocytosis. These results indicate that nanomaterials can be distributed beyond the initial site of exposure and accumulate in secondary organs, highlighting the importance of biodistribution studies in understanding the nanotoxicological outcomes.

On the basis of the evidence accumulated over this first decade, it was possible to conclude that nanostructures can accumulate in organs, induce cytotoxicity and trigger oxidative stress. Toxicity is associated with size, shape, composition, and functionalization, with smaller or persistent nanostructures being associated with a greater risk. It also became clear that exposure routes initially considered unlikely, such as the cutaneous route, are relevant from a toxicological perspective. In general, biodegradable or functionalized nanostructures tend to be less toxic,^41,42^ reinforcing the importance of characterizing and controlling the production process.

Throughout this first decade, nanotoxicology has evolved from exploratory studies, primarily focused on identifying potential risks, to more complex research aimed at understanding the relationship between nanomaterial properties and biological responses. Early studies focused mainly on assessing whether engineered nanomaterials could induce adverse biological effects, particularly in the respiratory systems. As evidence accumulates, research has increasingly focused on understanding how physicochemical properties, such as size, morphology, surface chemistry, and functionalization, influence biodistribution, persistence, and toxicity. This transition provided a foundation for future advances in risk assessment, including investigations of ADME processes, protein corona formation, chronic exposure, mechanistic toxicology, and predictive nanotoxicological models. Instead of considering nanoparticles only as a source of local toxicity, researchers have increasingly recognized their ability to cross biological barriers, accumulate in organs, and produce systemic effects.

In 2013, Keck and Müller^52^ proposed a classification system for nanostructures based on size and biodegradability, which may aid in categorizing them by their toxicological potential. Biodegradability is understood as the persistence of a substance within biological systems. In accordance with this framework, nanostructures can be divided into four distinct classes. Class I comprises biodegradable nanostructures larger than 100 nm, which are considered to exhibit low toxicity potential. Class II includes nonbiodegradable nanostructures larger than 100 nm. Class III encompasses biodegradable nanostructures smaller than 100 nm. Both Classes II and III are regarded as presenting a moderate risk of toxicity. Finally, Class IV includes nonbiodegradable nanostructures smaller than 100 nm, which are associated with high toxicity potential. Nanostructures smaller than 100 nm can more readily penetrate cells and tissues, leading to widespread biodistribution and an increased likelihood of adverse biological effects. Moreover, nonbiodegradable nanostructures tend to accumulate because of limited elimination, further increasing their potential toxicity. Both physicochemical properties and biological persistence are clearly critical determinants of toxicity.

Other nanotoxicological studies from the first decade, from 2005 to 2014, are presented in Table 1, which describe the nanomaterials evaluated, the biological models in which the nanostructures were applied, the procedures used to assess nanotoxicity, and the results of nanotoxicity testing. Table 1 presents important trends that characterized the first decade of nanotoxicological research. Most studies have focused on metallic nanoparticles, quantum dots, and carbon nanotubes, reflecting the growing technological interest in these materials. Heterogeneity is observed across test conditions, including doses, exposure routes, and biological models employed, ranging from microorganisms and isolated cell cultures to zebrafish and rodents, highlighting the absence of standardized testing strategies.^31,53^ This methodological diversity was characteristic of this period and contributed to inconsistencies across studies, reducing reproducibility and comparability between data sets and ultimately limiting the generation of evidence needed to support standardized risk assessments and regulatory frameworks.

**Table 1:** Nanotoxicological Studies in the First Decade, from 2005 to 2014^a^

the first decade of studies				
nanostructure	biological system (BSYS)	methodology	toxic effects	refs
TiO_2_ NP (66 and 950 nm, 44 μm) SiO_2_ NP (14 and 930 nm, 60 μm) ZnO NP (67 and 820 nm)	E. coli DH5α and B. subtilis CB310	liquid medium of a biological system containing the NPs (10–5000 ppm), 36 °C: 14 and 20 h	antibacterial activity increased with nanoparticle concentration; ZnO showed greater antibacterial activity than SiO_2_; B. subtilis was more susceptible than E. coli	(Adams et al. 2006)^129^
SWCNT (26 and 0.23 wt % iron)	RAW 264.7 macrophages	BSYS exposure to SWCNT suspensions (0.12–0.5 mg/mL), 37 °C: 1–2 h	iron content-dependent increase in oxidative stress (superoxide, nitric oxide) and cytotoxicity in alveolar macrophages, independent of nanotube diameter	(Kagan et al. 2006)^49^
Qd (565–655 nm)	pigs	topical application by flow through cells 62.5 pmol/cm^2^, 8 or 24 h	penetration of quantum dots limited to the outer skin layers (stratum corneum and epidermis) in intact skin	(Ryman-Rasmussen et al. 2006)^48^
Cu NP (80 nm)	wild-type zebrafish	BSYS exposure to Cu NP suspensions (0.25 and 1.5 mg/L)	concentration-dependent gill toxicity in zebrafish (0.25 vs 1.5 mg/L)	(Griffitt et al. 2007)^45^
silica NP (15 and 46 nm)	cultured human bronchoalveolar carcinoma-derived cells	BSYS exposure to silica NP suspensions (10 and 100 μg/mL)	dose-dependent cytotoxicity independent of particle size	(Lin et al. 2006)^127^
SWCNT (diameters: 1 nm, lengths between 300 and 1000) with DTPA	female BALB/c mice	injection of 200 μL of SWCNT suspensions (60 μg of SWCNT) in the BSYS via the tail vein	reduced systemic toxicity of DTPA-functionalized SWCNTs relative to nonfunctionalized controls	(Singh et al. 2006)^41^
Au NP (13.5 nm)	male ICR mice	oral administration, intraperitoneal, and tail vein injections of NP suspensions in the BSYS: 1100 μg/mL. oral administration of NP suspensions in the BSYS: 137.5–2200 μg/kg	route-dependent toxicity: greater adverse effects following oral and intraperitoneal administration compared to intravenous injection	(Zhang et al. 2010)^55^

a BSYS: biological system; NP: nanoparticle; Qd: quantum dots; SWCNT: single-walled carbon nanotube; RAW 246.7: mouse macrophage, Abelson murine leukemia virus-transformed; ICR: Institute of Cancer Research.

Furthermore, toxicological assessments have predominantly relied on acute exposure scenarios and conventional in vitro models with insufficient attention to chronic or environmentally relevant doses. Nanotoxicity was assessed by evaluating cytotoxicity, oxidative stress, antimicrobial effects, dermal penetration, and organism-level toxicity. Despite the variability, these studies have collectively demonstrated that nanotoxicity depends on factors beyond chemical composition.^54^ Particle size, surface functionalization, residual metals, exposure pathways, and biological contexts are fundamental to the observed toxicological responses.^41,49,55^ The need to develop and apply methodologies that account for the complexity of nanostructures, their interactions, and their physiological context has become evident.

### Advances in the Last Decade, from 2015 to 2024

Over the past decade, nanotoxicology has undergone significant development with a particular emphasis on describing the interactions of nanostructures with biological systems. Studies have increasingly focused on elucidating the molecular and systemic mechanisms of interactions between nanostructures and biological systems, including absorption, distribution, metabolism, and excretion (ADME) while also considering long-term biological impacts and environmental consequences.

The interdisciplinary nature of nanotoxicology, encompassing nanotechnology, toxicology, cell biology, environmental science, and health science, has driven innovation in both experimental models and analytical tools. Some of these innovations are summarized as follows.

### Toxicity Studies of Nanomaterials

#### Cytotoxicity Assays

Techniques for evaluating the toxicity of materials at the macroscopic scale are well-established but are underutilized in nanotoxicology. Additionally, some conventional methods, particularly in vitro cell-based tests, have been unable to yield consistent results on the nanotoxicology of materials because of their limitations and significant variability.^56^ Thus, traditional procedures for evaluating the toxicity of macromaterials have been adapted to assess parameters, such as oxidative stress, cellular internalization, and the molecular interactions of nanomaterials. Additionally, in recent years, new analytical methodologies have been developed and applied to nanotoxicology studies, expanding the range of available tools.

Among the conventional methods, cytotoxicity assays are a classical strategy for evaluating the adverse effects of nanomaterials on cell models. Commonly used assays include colorimetric or fluorometric reagents, such as 3-(4,5-dimethylthiazol-2-yl)-2,5-diphenyltetrazolium bromide (MTT) and resazurin.^57^ MTT assays quantify mitochondrial metabolic activity, and resazurin serves as a redox indicator that is reduced by viable cells. Both methods are simple, cost-effective, and widely used in the toxicological screening of nanomaterials.^58^ However, these methodologies have limitations for applications to nanostructures. Some nanostructures, especially metallic nanostructures, can directly interfere with optical signals, thereby compromising the accuracy of the readings.^59^ There is also the possibility of chemical interactions between nanomaterials and reagents, which can compromise the results.^60^

Thus, new methodologies, such as xCELLigence, have been used to assess the cytotoxicity of nanostructures. Instead of using dyes to determine cell viability, this technique is performed in real time using impedance-based electrode systems. In this way, the possible effects of interactions between the reagents and the tested nanostructures are avoided.^61^ The toxicity of various nanomaterials, including gold and silver nanostructures, carbon nanotubes, silica nanoparticles, and zirconium oxide nanoparticles, has been previously assessed via xCELLigence in different cellular models.^43–45^

In these cytotoxicity assays, three-dimensional (3D) cell culture models such as spheroids, organoids, and scaffold-based systems are gaining prominence. These are cell cultures performed in tissue-like microenvironments that aim to simulate the extracellular matrix and dynamic tissue conditions such as fluid flow.^62^ They exhibit greater similarity to real organs, including structural and functional properties as well as a longer lifespan, than 2D models do. Furthermore, their application does not depend on the ethical conditions imposed on animal models and is in accordance with the current guidelines for the replacement, reduction, and refinement of animal testing.^63^

This model has already been used to evaluate the toxicity of various nanostructures, including silver,^64^ silicon dioxide,^65^ titanium dioxide,^65^ and carbon nanotubes.^66^ Wiils et al. (2016)^67^ compared the 3D model with the 2D model for the toxicity assessment of silica nanoparticles in reconstituted skin tissue. In the 2D model, greater genotoxicity and reduced cell viability were observed compared with those in the 3D model receiving the same dose. These findings suggest that the cellular architecture and the skin’s collagen layer act as a barrier, preventing the nanostructure from reaching the cell. This evidence highlights the importance of incorporating three-dimensional models into nanotoxicological evaluation protocols, as the structural complexity of these systems enables more accurate predictions of nanoparticle behavior and effects in real biological tissues.

### Assessment of Oxidative Stress

Another way to assess the toxicity of nanomaterials is by assessing the oxidative stress. One of the most studied mechanisms is the use of reactive oxygen species (ROSs) to induce oxidative stress. Currently, techniques such as fluorescent probes are increasingly being used to assess oxidative stress and ROS production. They are light-sensitive molecules that emit fluorescence when excited by a radiation source, typically ultraviolet or visible light, and are used to detect and quantify biological and chemical processes within cells. In addition to being highly sensitive, they are noninvasive and allow for real-time monitoring.^56^

For example, Yang et al. (2010)^68^ reported a significant increase in ROS levels in A549 lung cells upon exposure to ZnO nanoparticles under UV light. L929 fibroblasts treated with Ag nanoparticles and Au nanoparticles also showed pronounced generation of ROS, particularly with silver, as quantified via 2′,7′-dichlorodihydrofluorescein diacetate (DCFH-DA).^69^ Additionally, human blood cells exposed to Ag nanoparticles demonstrated significant oxidative stress responses, as monitored via both DCFH-DA and DHE (dihydroethidium) probes.^70^

The production of ROS, such as superoxide (O^2–^), hydrogen peroxide (H_2_O_2_), hydroxyl radical (OH), and singlet oxygen (^1^O_2_), is often associated with the presence of transition metals on the surface of nanomaterials, such as silver (Ag), copper (Cu), iron (Fe), and zinc (Zn), which catalyze intracellular redox reactions,^71^ as shown in the following examples:

- Fenton and Haber–Weiss reactions (Fe^2+^ + H_2_O_2_ → OH), which result in the production of the hydroxyl radical, an extremely reactive and cytotoxic species.^71,72^

- Photocatalytic activity, especially for TiO_2_ under UV radiation, leads to the formation of O^2–^, H_2_O_2_, and ^1^O_2_.^73^
- Interference with the mitochondrial electron transport chain leads to the formation of O^2–^, mainly at complexes I and III.^74^
- The activation of enzymes such as nicotinamide adenine dinucleotide phosphate oxidase (NADPH oxidase) leads to the production of extracellular superoxide during the phagocytosis of nanoparticles.^75^

A direct consequence of increased ROS production is the depletion of the cellular antioxidant system, particularly glutathione (GSH), which serves as a line of defense against oxidative damage. GSH depletion is a classical marker of oxidative stress and has been observed, for example, after exposure of human liver cells (HepG2) to CuO and Ag nanoparticles.^76^

Moreover, ROS activate intracellular signaling pathways such as NF-κB, MAPK (p38, JNK, and ERK), and proapoptotic proteins such as p53 and caspases, contributing to inflammatory processes, mitochondrial damage, deoxyribonucleic acid (DNA) fragmentation, and apoptosis.^77,78^

Although ROS generation is frequently discussed at the molecular level, its toxicological relevance lies in its ability to link nanoparticle reactivity to tissue- and organism-level damage. The activation of inflammatory and apoptotic pathways, along with oxidative damage, has been associated with neurotoxicity, cardiotoxicity, hepatotoxicity, nephrotoxicity, pulmonary inflammation, and fibrosis across various nanomaterial exposure models. Therefore, oxidative stress should be viewed not only as a biomarker but also as a key mechanism linking the physicochemical properties of nanomaterials to adverse biological effects.^79^

Recent studies have shown that the surface chemistry of nanostructures and the composition of the protein corona influence ROS generation and subsequent toxicity (Table 2). In terms of silver, zinc oxide, and gold nanostructures, corona-free particles typically exhibit faster ion dissolution, greater hydroxyl radical production, and more intense activation of inflammatory signaling. Conversely, the adsorption of serum proteins (e.g., HSA and HDL) often reduces ROS production, stabilizes the particle charge, and partially attenuates mitochondrial damage or cytokine induction.

**Table 2:** Protein Corona-Dependent Modulation of ROS Generation and Cytotoxicity by Engineered Nanomaterials

nanomaterial	corona condition	observed effect on ROS/mechanisms	impact on toxicity	refs
AgNPs (40 nm, citrate-stabilized)	without corona	higher Ag^+^ dissolution rate (≈5–6 times more than protein-coated). Possible ROS generation linked to oxidative stress	lower relative toxicity. HepG2 cells showed higher viability compared to corona-coated AgNPs	(Park et al. 2023)^130^
AgNPs (40 nm)	with corona (HSA or gHSA)	reduced Ag^+^ dissolution but increased NP-specific toxicity (e.g., membrane and mitochondrial damage, ROS)	∼2-fold increase in toxicity compared to uncoated AgNPs. No significant difference between HSA and gHSA	
AgNPs (20 nm, citrate-suspended)	without corona	readily taken up by RLE and RAEC cells. Induced cytotoxicity and inflammatory response (IL-6 expression)	dose-dependent cytotoxicity and inflammation via SR-BI receptor	(Shannahan et al. 2015)^82^
AgNPs (20 nm)	with corona (HSA, BSA, HDL)	HSA/BSA: reduced Ag^+^ dissolution and reduced uptake. HDL: increased dissolution and uptake via SR-BI. Overall, PCs altered charge and internalization	HSA/BSA reduced toxicity. HDL enhanced inflammation	
AgNPs (cubic/quasispherical, PVP)	without corona	higher Ag^+^ dissolution and Ag_2_S formation	high cytotoxicity in macrophages; TNF-α and MIP-2 induction	(Miclăuş et al. 2016)^84^
with corona (hard/soft, FBS)	with corona (hard/soft, FBS)	hard corona trapped Ag^+^; soft corona reduced sulfuration	reduced cytotoxicity and inflammatory response	
TiO_2_ nanotubes (∼10 nm diameter)	without corona	potent hydroxyl radical generation. ROS proportional to exposed surface	significant phototoxicity at high concentrations (≥1000 μg/mL)	(Garvas et al. 2015)^85^
TiO_2_ nanotubes (∼10 nm diameter)	with serum proteins (FBS)	proteins coat the surface and scavenge radicals. Reduction in ROS: ∼80%	phototoxicity is largely prevented; low toxicity even under UV	
ZnO NPs (∼29 nm)	without corona	strong ROS generation and higher dissolution are linked to oxidative stress	HepG2 viability dropped sharply (≤20% at ≥ 50 mg/L)	(Ying et al. 2015)^131^
ZnO NPs (∼29 nm)	preformed protein corona (FBS, 24 h)	stable protein layer reduced ROS generation and Zn ion release	high cell viability (85–95%) even at 100 mg/L	
AuNP (40–80 nm, BPEI/LA/PEG)	without corona	increased ROS/RNS, inhibition of CYP1A2, 2C9, 3A4	high hepatocyte cytotoxicity	(Choi et al. 2017)^83^
AuNP (40–80 nm, BPEI/LA/PEG)	with HSA/HP	HSA reduced uptake and ROS (protective). HP partially reduced ROS, but BPEI toxicity persisted	HSA reduced uptake and ROS (protective). HP partially reduced ROS, but BPEI toxicity persisted	

These findings highlight that oxidative outcomes cannot be interpreted solely based on intrinsic nanostructure properties; protein corona formation alters redox behavior. Taken together, the studies summarized in Table 2 demonstrate that the protein corona cannot be considered just a coating. It can alter dissolution kinetics, cellular uptake, inflammatory signaling, and ROS generation. This behavior explains why the same nanomaterial can exhibit a different toxicological profile depending on the biological environment in which it is dispersed.^80–82^

In most of the studies presented in Table 2, protein corona formation reduced ROS (reactive oxygen species) generation, ionic dissolution, cellular uptake, and inflammatory responses, thereby reducing cytotoxicity. This protective effect was observed for AgNPs, TiO_2_ nanotubes, ZnO nanoparticles, and AuNPs under specific biological conditions.^82–84^ Particularly noteworthy are the approximately 80% reduction in ROS generation reported for serum protein-coated TiO_2_ nanotubes and the marked improvement in HepG2 cell viability following protein corona formation with ZnO nanoparticles.^85^

However, in some cases, such as with citrate-stabilized AgNPs, protein adsorption decreased Ag^+^ dissolution while increasing the specific toxicity of the nanoparticles, resulting in approximately twice the cytotoxicity of uncoated particles. These observations indicate that the toxicological consequences of corona formation depend not only on its presence but also on its composition, stability, and influence on cellular uptake pathways.^10,80,82^ These findings suggest that the biological identity acquired by nanomaterials after contact with physiological fluids can be just as important as their original physicochemical characteristics in determining toxicological outcomes.^80^

### Physicochemical Properties, Absorption, and Distribution

The absorption and biodistribution of nanomaterials are closely related to their physical and chemical properties, such as size, shape, solubility, surface composition, and degree of agglomeration. These factors determine the route of entry into the body and subsequent biological destinations. Exposure to nanomaterials in the human body occurs mainly through inhalation, ingestion, dermal contact, and intravenous injection. Once inside the body, nanomaterials can cross important biological barriers, including the pulmonary epithelium, gastrointestinal mucosa, and blood–brain barrier. These processes occur via mechanisms such as passive diffusion, active transport, and endocytosis.^86,87^

The organs most frequently involved in the accumulation of nanomaterials are (i) the liver, owing to its role in particle clearance via the mononuclear phagocyte system; (ii) the kidneys, because of their involvement in filtration and excretion; (iii) the spleen, which contains abundant phagocytic cells; and (iv) the brain.^86,87^ Brain damage is related to the proven ability of nanostructures to cross the blood–brain barrier and enter the brain and other parts of the nervous system.^88^ The neurotoxicity of different nanostructures, such as silver^60^ and zinc oxide,^61^ has already been documented. Recordati et al. (2016)^89^ evaluated the toxicological effects of silver nanostructures in rats and assessed the influence of particle size and coating presence on toxicity and nanoparticle distribution in lung, liver, kidney, spleen, and brain tissues. The highest silver concentrations were observed in the spleen and liver, followed by those in the lung, kidney, and brain, with maximum concentrations of 121.7 μg/g in the spleen and 78.2 μg/g in the liver for particles smaller than 10 nm. In the studied organs, smaller nanostructures (10 nm) accumulated in greater quantities than larger particles did (40–100 nm) and caused more intense toxic effects.

Considering the growing interest in understanding how nanostructures are distributed and eliminated from the body, Poon et al. (2019)^90^ investigated the hepatobiliary route in mice as an alternative to renal excretion, especially for large nanostructures (above the glomerular filtration capacity) or nonbiodegradable nanostructures. Using gold nanoparticles as a model, the authors demonstrated that the kidneys predominantly eliminate particles smaller than approximately 5.5 nm, while biodegradable nanocarriers or larger structures can be degraded and return to systemic circulation.

On the other hand, nanostructures with hydrodynamic diameters larger than the glomerular filtration limit (∼5.5 nm) tend to evade efficient renal elimination and accumulate in organs rich in phagocytic cells, such as the liver, spleen, and lymph nodes.^91^ This prolonged retention may result in chronic inflammation, oxidative stress, and cellular dysfunction. The hepatobiliary route provides a compensatory excretion pathway but is slower and limited, especially for materials such as gold, TiO_2_, or specific synthetic polymers. Biopersistence can also be influenced by changes in particle surface properties during circulation, such as corona formation, interactions with enzymes, and the acidic pH of cellular compartments, which can affect the stability, reactivity, and solubility.^92^

Notably, when nanomaterials interact with biomolecules, enzymes, physiological fluids, and cellular compartments in living organisms, they can change their surface properties, aggregation state, degradation profile, and biomolecular corona composition, collectively influencing their biological interactions. These processes influence biodistribution, persistence, elimination, and, ultimately, the toxicological outcomes observed in exposed organisms. Recent studies have emphasized that understanding the long-term fate of nanomaterials requires considering not only their original physicochemical characteristics but also the dynamic transformations that they undergo throughout their life cycle.^93^

Sousa et al. (2023)^94^ demonstrated that orally administered polyvinylpyrrolidone (PVP)-stabilized silver nanoparticles (AgNPs) induced intestinal inflammation in mice and were predominantly excreted via feces, despite their systemic distribution. Similarly, a study by Rosário et al. (2022)^95^ revealed that compared with larger particles, AgNPs administered intratracheally in mice presented a size-dependent distribution of 5 nm and were retained longer in organs such as the liver, spleen, and kidneys, with both urinary and fecal elimination routes involved.

A study comparing gold nanoparticles (AuNPs), nanodiamonds, and carbon quantum dots revealed distinct tissue accumulation: nanodiamonds showed greater cardiac accumulation, whereas AuNPs were concentrated in the lungs, and carbon dots were concentrated in the kidneys.^96^ In neonatal rats, Xu et al. (2023)^97^ reported that polymeric nanoparticles accumulate preferentially in the liver and spleen, with surfactant properties significantly altering biodistribution and excretion kinetics.

These processes should not be viewed as independent events. The extent of absorption influences biodistribution patterns, whereas surface transformations occurring during circulation can modify the organ accumulation and elimination pathways. Consequently, toxicological outcomes result from the dynamic interactions among absorption, distribution, biotransformation, persistence, and excretion.^80,92^ Since many of these processes are influenced by surface modifications that occur after nanomaterials enter biological systems, understanding the roles of coatings and protein corona formation has become a central aspect of nanotoxicological research.

### Presence of Coatings and Protein Corona

Over time, numerous studies have investigated the addition of coatings, ligands, or additives to nanostructures and their potential toxic effects. Typically, this type of association affects how nanostructures interact with the organisms. Coatings or ligands with greater biocompatibility facilitate the recognition of nanostructures by biological systems, thereby reducing their toxicity. Intentional changes can modify the toxic effects of nanostructures, whereas environmental interactions can also alter these compounds and their biocompatibility.

According to Ozcakir (2023),^98^ a promising alternative to mitigate toxic effects is the incorporation of nanostructures, such as proteins, polysaccharides, and lipids derived from plant or animal sources, which are considered renewable, biodegradable, nontoxic, and environmentally safe, into biopolymers. In addition to these intentional surface modifications, interactions that occur after exposure to biological fluids can also alter the nanomaterial behavior. Among these acquired surface modifications, protein corona formation has become one of the most extensively investigated because of its profound influence on biological interactions.^80^ The protein layer of the corona spontaneously adsorbs onto the surface of nanostructures upon contact with biological fluids such as blood plasma. As a result, their physicochemical properties are altered, affecting their absorption, blood circulation, biodistribution, metabolism, and toxicity.^99^

The reduction in AuNP toxicity caused by the formation of an induced protein corona was demonstrated by Arya et al. (2022)^100^ in a study with *Allium* cepa root meristematic cells. According to Pourali et al. (2022),^101^ the formation of a protein corona reduces nanostructure toxicity by protecting it from immune system cell attack.

To better understand the mechanisms of protein corona formation and composition, various analytical techniques have been explored. Ex situ methods, which involve separating protein-bound nanostructures for subsequent characterization, and in situ methods, which analyze nanostructures dispersed in physiological media, are widely used. Typically, transmission electron microscopy and atomic force microscopy are applied to determine the size of the formed complexes. Moreover, polyacrylamide gel electrophoresis and liquid chromatography coupled with mass spectrometry (LC–MS/MS) are used to identify and quantify the proteins involved.^81^

Shannahan et al. (2015)^82^ evaluated the effect of the protein corona on the interaction of AgNPs with mouse macrophages. The nanostructures were contacted (i) with multiple proteins in a medium containing 10% fetal bovine serum to form a complex protein corona or (ii) with a single protein, bovine serum albumin, to create a simple protein corona. The presence of the protein corona stabilized the nanostructures by inhibiting the release of Ag^+^ ions and altering their uptake by mouse cells, possibly reducing their toxicity potential. These findings highlight the complexity of predicting toxic effects as the protein corona composition can vary depending on the characteristics of the nanostructures and the conditions of the biological environment.

Remarkably, in a more in-depth analysis of studies on the protein corona, we observed that its effect on the nanostructure toxicity is not always positive. A reduction in toxicity occurs when it promotes stabilization and reduces ionic dissolution, prevents direct contact with the cell membrane, or reduces its ability to generate an inflammatory response. However, in some cases, the formation of the protein corona can increase the dissolution of metallic nanostructures, expose reactive sites, promote exacerbated cellular internalization, or exhibit immunogenic activity, all of which can contribute to increased toxicity.^80^

#### Environmental and Agricultural Toxicity

In parallel with studies evaluating nanotoxicity in mammals and microorganisms, studies on the toxicology of nanostructures in plants have been conducted. The application of nanostructures in plant cultivation is based on their unique characteristics such as high permeability, biocompatibility, biodegradability, optical properties, and adsorption capacity. In this way, nanostructures can be used as carriers of nutrients and pesticides, contributing to the growth and development of plants, as well as for the screening and quantification of pesticides and other pollutants.^102,103^

In the first review on the subject, Mehrian and De Lima (2016)^104^ addressed the genotoxicity of nanostructures in plants, and according to them, the mechanisms involved are either direct or indirect. In terms of direct genotoxicity, nanostructures can cross cellular and nuclear membranes (via diffusion or endocytosis) and interact with DNA either mechanically or chemically. Indirect genotoxicity results from interactions between nanostructures and nuclear proteins, oxidative stress, and reduced DNA repair function. The authors stated that genotoxicity increases with decreasing particle size, increasing concentration, and increasing exposure duration. Particularly, this inverse relationship between particle size and genotoxicity parallels findings independently reported in mammalian and fish models involving silver and gold nanoparticles,^89,105,106^ suggesting that particle size may function as a cross-kingdom predictor of nanotoxicological outcomes, largely independent of the biological system under study.

On this basis, subsequent work broadened its scope to include a wider range of nanomaterials and plant species. In 2021, Jogaiah et al.^107^ compiled studies that evaluated the toxicological effects of various metallic, polymeric, and carbon nanostructures on multiple plant species. Cytotoxic and genotoxic effects, including excessive production of free radicals, interference with the electron transport chain, DNA damage with inhibition of protein synthesis, impacts on seed germination, and accumulation in tissues, are considered. According to the authors, it is necessary to standardize test parameters, such as nanostructure size, concentration, and dosage, to ensure comparability of the results. The level and duration of exposure, interactions with the environment, and life cycles may also be considered when standardizing test parameters. These considerations are relevant to agricultural applications, in which engineered nanomaterials are intentionally applied to cropping systems. Therefore, toxicity assessment is essential not only to ensure plant safety but also to minimize unintended effects on the soil organisms, surrounding ecosystems, and food chain.

Research on nanotoxicology has also been conducted to assess its implications for aquatic ecosystems. Metallic nanostructures can penetrate the tissues and cells of aquatic organisms and accumulate in the food chain, potentially posing risks to human health. Additionally, nanostructures of commercial polystyrene are transported through the food chain of aquatic ecosystems from algae to zooplankton and fish. Metabolic changes and alterations in feeding behavior were observed as negative impacts on fish following the ingestion of organisms contaminated with nanostructures.^108^ Consistent with these observations, Kerin et al. (2023)^109^ published a review that compiles the exposure routes and toxicological effects of metallic nanostructures on aquatic organisms and environments, considering gold, silver, copper oxide, and aluminum oxide nanostructures. According to these authors, the toxicity of nanostructures is related to their accumulation in organisms and dissolution in water, both of which depend on the particle size and surface characteristics. These studies that address nanotoxicological effects in aquatic systems are very informative as many waste streams containing nanomaterials can reach freshwater or marine systems.

Given their environmental impact, microplastics and nanoplastics (M-NPLs) are recognized as significant pollutants. They can originate from a variety of sources including product manufacturing, biomedical applications, textiles, industrial pollutants, urban transportation, laundry, and landfills. Therefore, humans and the environment are increasingly being exposed to them.^110^ The accumulation and toxicity of polystyrene nanoparticles administered to mice are dependent on their size and concentration. Smaller particles (50 nm) at a dosage of 10 mg/kg body weight resulted in greater accumulation in the liver, spleen, and intestine in addition to alterations in liver, kidney, and cardiac function.^111^

Although early studies on the environmental implications of nanomaterials focused on their direct toxicity to individual species, recent research has increasingly demonstrated that environmental transformations strongly influence their fate and ecological risks. Phenomena such as dissolution, sulfidation, corona formation, and aging alter the physicochemical properties of nanomaterials prior to their interaction with organisms and, consequently, their bioavailability, uptake, and ecological effects.^112^ Thus, environmental nanotoxicology has evolved toward approaches that consider the environmental fate, trophic interactions, and long-term sustainability.^113^

The importance of considering environmentally transformed nanomaterials is further highlighted by studies investigating trophic transfer. Environmental transformations influence the movement of nanomaterials through food chains, meaning that ecological risk depends not only on the intrinsic properties of the original nanomaterials but also on the forms they take under environmentally relevant conditions.^112,113^ For instance, weathered carbon nanotubes have been shown to transfer from algae to *Daphnia magna*.^114^ In contrast, silver nanoparticles and their transformation products are transferred along the *Daphn-brafish* food chain, with their biodistribution and biomagnification influenced by environmental transformation processes.^115^

#### Predictive Toxicity Models

Knowledge about nanotoxicology has evolved through studies evaluating various nanostructures in multiple models and environments. However, given the variety of factors that can influence organisms’ responses to these compounds, many studies have failed to reach general conclusions or meet reproducibility standards. Artificial intelligence (AI) and machine learning (ML) have been increasingly applied in these processes. Their ability to analyze large sets of complex data and extract relevant information makes them promising for better understanding the potentially toxic effects of nanoscale materials. This growing analytical capacity has paralleled the broader expansion of the field, as reflected in its scientific output over time. A search of the Web of Science (2026)^116^ using the term “nanotoxicology” revealed that publications increased steadily from 2004 (*n* = 2), peaking between 2014 and 2016 (*n* = 387), followed by a decline through the 2023–2024 period (*n* = 137–162) and a partial recovery in 2025 (Figure 3). This pattern was corroborated by a complementary search in PubMed (2026),^117^ which revealed a similar trajectory. Rather than indicating a reduction in research activity, this observation likely reflects diversification into specialized subfields and emerging phenomena including nanosafety, safe-by-design approaches, regulatory risk assessment, nanoecotoxicology, and AI-based toxicity prediction, all of which are addressed throughout this review. Several works applying artificial intelligence and machine learning to nanotoxicological data are presented in Table 3.

**Figure 3:**
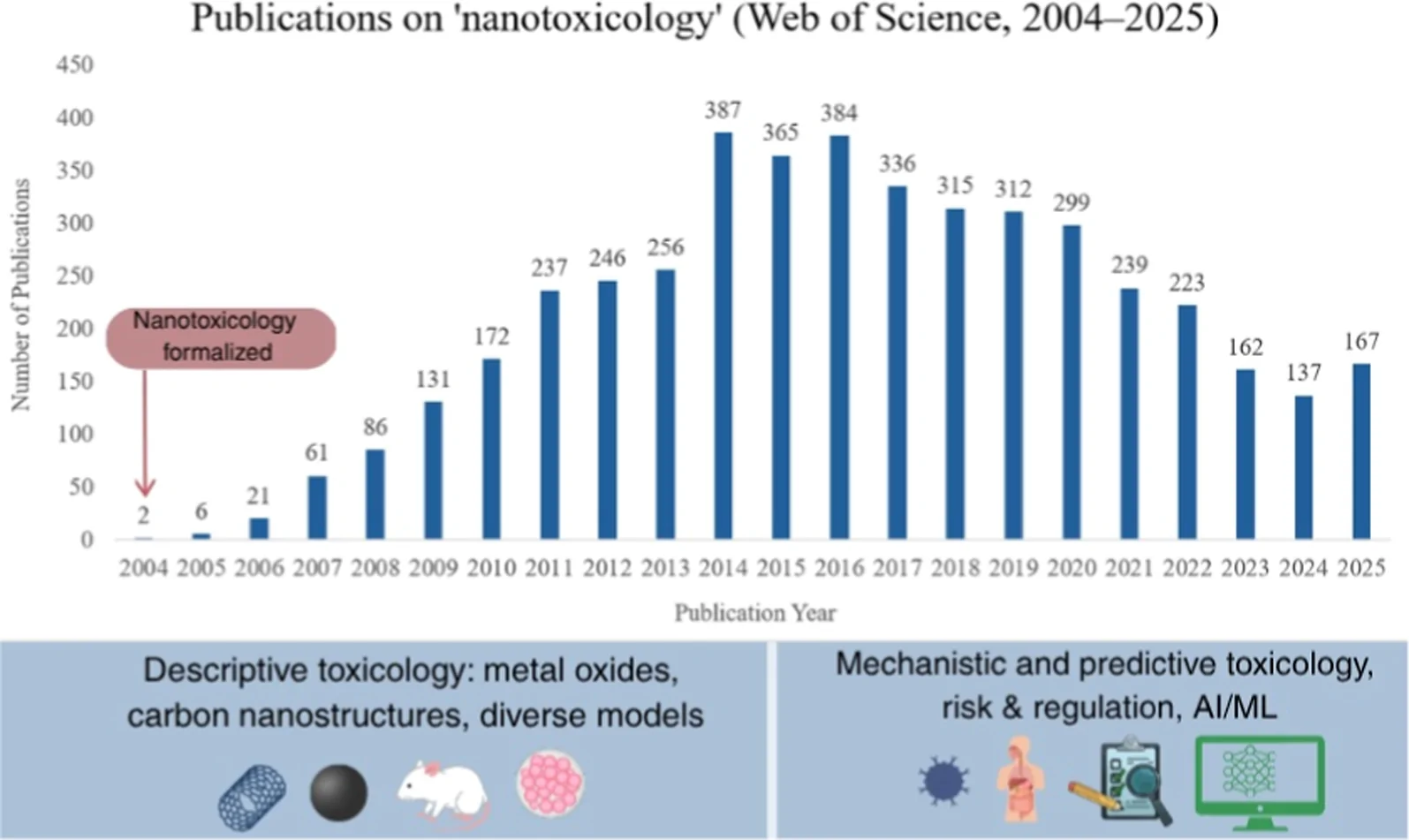
Evolution of nanotoxicological studies over time. Bars represent the annual number of records retrieved using the term “nanotoxicology” in Web of Science (accessed June 2026). Shaded bands indicate the first and second decades of nanotoxicological research, characterized respectively by predominantly descriptive approaches and by increasing mechanistic, regulatory, and computational sophistication.

**Table 3:** Examples of AI/ML-Based QSAR and QSTR Models for Predicting Nanomaterial Cytotoxicity and Oxidative Stress Potential

nanomaterials	biological model	method (AI/ML)	main findings	refs
fullerene (C60), graphene nanosheets (GN), graphene oxide (GO), single-walled carbon nanotubes (SWCNTs), multiwalled carbon nanotubes (MWCNTs), and their binary mixtures	Scenedesmus obliquus	nano-QSAR with ML algorithms: C4.5 Decision Tree, SVM, ANN, Naive Bayes, KNN, Logistic Regression, Random Forest, AdaBoost. Regression: Decision Tree, Random Forest, Gradient Boosting, AdaBoost	ML-based nano-QSAR models accurately predicted oxidative stress (ROS generation). Zeta potential was the most relevant descriptor	(Qi and Wang 2025)^132^
metal oxide ENMs (ZnO, Ag, SiO_2_, CeO_2_, etc.)	human lung carcinoma cells (A549)	nano-QSTR, Decision Tree, Random Forest, Extra-Trees	Extra-Trees model achieved the best predictive performance (*R* ^2^ train = 0.95; *Q* ^2^ ext = 0.79). Smaller ENM diameter increased cytotoxicity; PEG coating reduced cytotoxicity	(Meneses et al. 2023)^133^
nano-TiO_2_ mixed with eight heavy metals (Cd, Zn, Cu, Ni, Pb, Mn, Sb, Co)	human renal cortex proximal tubule cells (HK-2)	QSAR with Random Forest +K-means clustering	RF outperformed AdaBoost. Clustering improved model accuracy (*R* ^2^ test up to 0.95; RMSE = 0.06). Identified adsorption energy and hardness as key descriptors	(Sang et al. 2022)^134^
metal-oxide nanoparticles (Al_2_O_3_, CuO, Fe_2_O_3_, TiO_2_, ZnO)	generalized in silico data set (no specific cell line)	ML models: Logistic Regression, Random Forest, SVM, Neural Networks; integrated into NanoTox ensemble	parsimonious feature set (9 descriptors) achieved high accuracy (>96%). Dose and exposure time were key predictors. NanoTox ensemble reached ∼98% balanced accuracy	(Subramanian and Palaniappan 2021)^135^
oxide NPs (7 types; physicochemical, toxicological, quantum attributes)	in vitro data (S2NANO; 14 cell lines)	RF, NN, BN, kNN, and SVM, ensembles (Voting, Copeland Index)	RF and NN were the best base classifiers. ensembles LIR: LWL-IBk-RF outperformed base. More robust with missing data	(Furxhi et al. 2019)^136^
binary mixtures of metal oxide NPs (CuO, ZnO, TiO_2_, ZrO_2_, Al_2_O_3_, Fe_2_O_3_, SiO_2_)	E. coli (cytotoxicity assays, 22 binary combinations)	QSAR models with SVM and NN	NN-QSAR models (esp. N31) outperformed SVM and classical mixture models (CA, IA). Descriptors ΔHme^+^ and ΔHsf were key predictors. Models were reliable for toxicity prediction of ENP mixtures	(Zhang et al. 2023)^137^
SiO_2_, ZnO, Fe_3_O_4_ (plus tests on Co_3_O_4_, Mn_2_O_3_)	E. coli K12 TG1 (luminescence inhibition assay)	light GBM Regressor & classifier (Optuna-tuned). compared with RF, XGB, and Extra-Trees	accurate models (*R* ^2^ ≈ 0.99, Acc ≈ 0.98). NP size and concentration most relevant. ZnO is the most toxic. SiO_2_ stimulatory	(Khokhlov et al. 2024)^138^
metal oxide ENPs (CuO, ZnO, TiO_2_, Fe_2_O_3_, Al_2_O_3_, etc.)	multiple cell lines (in vitro cytotoxicity)	ensemble QSAR with kNN, SVM, DT, RF, and XGB	ensemble model improved prediction accuracy over individual ML methods. Key descriptors linked to solubility and surface chemistry	(Hosni et al. 2025)^139^
metal oxide NPs (ZnO, TiO_2_, CuO, Fe_2_O_3_, Al_2_O_3_, SiO_2_)	multiple mammalian cell lines (in vitro cytotoxicity)	QSAR with RF, SVM, and ANN	RF gave the best performance. Descriptors related to size, surface area, and band gap were most relevant for toxicity	(Wang et al. 2025)^140^

In recent years, the integration of AI and ML has also enabled the rapid screening of nanomaterials by linking physicochemical descriptors to toxicological outcomes. Instead of relying solely on empirical assays, the QSAR/QSTR models infer patterns of cytotoxicity and ROS generation across different particle types. Table 3 presents representative studies (2019 to 2025) that demonstrate that algorithms such as random forests, support vector machines, and gradient-boosted ensembles achieve high predictive accuracy; identify key descriptors (e.g., particle size, ζ-potential, and band gap); and highlight dose and exposure time as critical drivers of response. Collectively, the studies summarized in Table 3 indicate a transition from descriptive nanotoxicology to predictive modeling. Nevertheless, the reported accuracy of many models should be interpreted with caution, as data set heterogeneity, limited external validation, and model interpretability remain important challenges.

Despite these promising advances, several limitations must be considered when interpreting the performance of AI- and machine learning (ML)-based nanotoxicology models. Conventional nano-QSAR approaches are generally developed under the assumption of complete and homogeneous data sets. However, nanotoxicological data are often incomplete and generated under heterogeneous experimental conditions, including differences in nanomaterial characterization, exposure protocols, biological models, and toxicological end points. Furthermore, nanomaterials can interfere with conventional toxicity assays, introducing experimental artifacts that may lead to a substantial overestimation or underestimation of toxicity. Consequently, AI and ML models inherit many of the limitations of the data sets used for training and validation, reinforcing the principle that their predictive performance cannot exceed the reliability of the underlying experimental evidence.^118–120^

Therefore, increasing the reliability of AI- and ML-based nanotoxicology models depends not only on advances in computational methods but also on the availability of high-quality, standardized, and interoperable experimental data sets. In this regard, adopting the FAIR (findable, accessible, interoperable, and reusable) data principles has been identified as crucial for improving data comparability and facilitating the integration of results generated across different laboratories. Comprehensive metadata describing the physicochemical characterization of nanomaterials, exposure conditions, dosimetry, and biological end points are essential for developing robust predictive models and enhancing their applicability across diverse experimental contexts. There is a clear need for harmonized reporting standards, rigorous data curation practices, and interoperable databases that support AI applications that are reproducible and relevant to regulatory purposes in nanotoxicology.^31,54,120,121^

Even when high-quality data sets are available, rigorous model evaluation remains essential for establishing the reliability of artificial intelligence (AI)- and machine learning (ML)-based tools. High predictive metrics, such as *R* ^2^, should be interpreted with caution, as they can be artificially inflated by overfitting and may not reflect model performance on independent data sets. Consequently, external validation has become a critical step for assessing model robustness and generalizability, particularly because relatively few published nano-QSAR and AI-based models have been validated using independent data sets generated by external laboratories.^122^ In addition to external validation, improving model transparency remains essential for regulatory acceptance, as the “black-box” nature of many machine learning algorithms limits the interpretability of their predictions. In this context, explainable artificial intelligence (XAI) has emerged as a promising approach for improving model transparency and interpretability, thereby facilitating the future integration of AI-based approaches into regulatory decision-making.^123^ Furthermore, Bayesian frameworks and weight-of-evidence (WoE) approaches have been proposed to explicitly incorporate predictive uncertainty and support transparent, evidence-based regulatory risk assessment.^124^

These advances reinforce the notion that nanotoxicity is a dynamic process driven by continuous interactions between nanomaterials and biological and environmental systems. Instead, toxicity results from the dynamic interactions among the exposure pathway, physicochemical transformations in biological or environmental media, and mechanisms that influence absorption, distribution, metabolism, and excretion. The formation of the protein corona and environmental transformations cannot be separated from the dissolution kinetics, redox reactivity, immunological activation, or biodistribution profiles. Future advances in nanotoxicology will likely depend on the ability to incorporate nanomaterial biotransformation, persistence, degradation, and recycling processes into predictive frameworks. Because nanomaterials undergo continuous physicochemical and biological modifications throughout their life cycle, their long-term behavior cannot be reliably predicted solely from their pristine characteristics. Integrating life-cycle transformations with experimental and computational toxicology approaches may substantially improve the prediction of chronic effects, organ-specific accumulation, biodistribution, and environmental risks associated with emerging nanomaterials.^93^

### Current Challenges and Research Gaps

Hu et al. (2016)^125^ raised pertinent questions about the disparity between various nanotoxicological studies and the actual conditions of nanomaterial application. Many nanotoxicological studies are conducted in culture media, saline solutions, or buffered solutions that do not accurately reflect the complex biological environments of organisms. Although there are no precise estimates, environmental concentrations are generally expected to be low and may occur at trace levels, given the low doses at which they are used. These concentrations are substantially lower than those typically considered in toxicity studies of conventional materials with the same chemical composition. Moreover, determining the biologically relevant dose remains a major challenge in nanotoxicology because the nominal exposure concentration does not necessarily reflect the internal dose reaching tissues and cells, which depends on uptake, biodistribution, and biological interactions. Most toxicological studies are conducted over a short period, whereas organisms’ actual exposure to these compounds is typically long-term. Thus, the life cycle of organisms must be considered when determining the exposure time. Chronic exposure to nanomaterials enables the evaluation of reversibility and adaptation to nanotoxicity. The possibility of long-term retention of nanostructures in ecosystems raises significant ethical questions, as their persistence and potential bioaccumulation in food chains pose poorly understood risks that could harm future generations.

As previously presented, there is still no standardization of test protocols, making comparisons, reproducibility, and accurate conclusions difficult.^53^ These limitations not only affect the interpretation of toxicological findings and the development of predictive models but also hinder regulatory convergence by restricting the establishment of harmonized testing strategies and globally accepted exposure limits. In addition, the transformations that nanomaterials undergo in the environment are very complex, and it is not yet possible to predict them under different application conditions. They are usually not reproduced in studies, especially in vitro, which limits their validity.^125^

Table 4 presents a selection of nanotoxicological studies from the second decade, spanning from 2015 to 2024, that describe the nanomaterials evaluated, the biological models in which the nanostructures were applied, the procedures used to assess nanotoxicity, and the results of the toxicity of the nanostructures. Compared with the first decade, during which research focused primarily on carbon nanostructures for medical applications, studies conducted between 2015 and 2024 became more diverse, with greater interest in the molecular mechanisms of toxicity and environmental impacts. It is worth noting that size-dependent toxicity emerged as one of the most reproducible findings in independent studies conducted during this period: compared with larger nanostructures (≥50–100 nm), smaller silver and gold nanoparticles (10–20 nm) exhibited greater tissue retention, altered biodistribution, and genotoxicity, regardless of differences in animal models, routes of administration, or dosages.^89,105,106,126^ This convergence suggests that particle size, unlike many other experimental variables, acts as a robust and reproducible predictor of nanotoxicological outcomes, which is consistent with the cross-kingdom size-dependence pattern noted earlier in this review in the context of plant genotoxicity.^104^ This pattern contrasts with the findings of early first-decade studies, such as Lin et al. (2006)^127^ (Table 1), who reported dose-dependent cytotoxicity independent of particle size (15 vs 46 nm). This discrepancy may reflect methodological refinements in dosimetry and characterization over the following decade, rather than a true absence of size dependence in early nanomaterials. Beyond this specific pattern, however, establishing definitive conclusions about the toxic effects of nanostructures and identifying the most suitable methodologies for their assessment remain challenging owing to the substantial variability in experimental approaches across studies. Even when the same type of nanostructure is evaluated, differences in testing protocols, biological models, and exposure conditions hinder the comparability and standardization of results.

**Table 4:** Nanotoxicological Studies from the Second Decade, Spanning from 2015 to 2024^a^

the second decade of studies				
nanostructure	biological system (BSYS)	methodology	toxic effects	refs
Ag NP (20 and 200 nm) TiO2 NP (21 nm)	male WT and Ogg1 KO mouse	intravenous application of NP suspensions in the BSYS dose: 5 mg/kg	greater DNA damage and altered gene expression associated with smaller particle size (20 nm) relative to larger particles (200 nm)	(Asare et al. 2016)^106^
Ag NP (10, 40, and 100 nm) (citrate- or polyvinylpyrrolidone-coated)	male CD-1(ICR) mice	intravenous application of NP suspensions in the BSYS dose: 5 mg/kg	size-dependent organ accumulation, with greater retention of 10 nm particles	(Recordati et al. 2016)^89^
Au NP [spheres (10 and 50 nm), rods (60 × 30 nm), stars (average 55 nm longest tip-to-tip distance)	male CD-1 mice	intravenous application of NP suspensions in the BSYS dose: 1.1 × 10^11^ NPs/mL (140 μL)	size- and shape-dependent organ biodistribution	(Talamini et al. 2017)^105^
Ag NP (12.3 nm)	Sprague–Dawley male rats	intragastric application of NP suspensions in the BSYS single dose: 2000 mg/kg multiple doses: 250 mg/kg	no overt systemic toxicity; accumulation detected in liver and kidneys	(Hendrickson et al. 2016)^141^
Ag NP (hybrid lipid-coated) (20–100 nm)	wild-type tropical 5D zebrafish (Danio rerio)	zebrafish were incubated in a suspension of AgNP (0.25 and 12 mg/L)	greater toxicity in zebrafish associated with smaller particle size and higher concentration (0.25 vs 12 mg/L)	(Cunningham et al. 2021)^126^
IO NP (differently charged: negative, neutral, and positive) (14.58 nm)	A549 (lung HCC) Huh-7 (liver HCC) SH-SY5Y (neural HCC) BALB/c mice	intravenous injection of NP suspensions in the BSYS doses: 2 mg/kg and 10 mg Fe/kg of animal	no significant toxicity was observed	(Woo et al. 2021)^142^
TiO2 NP (cotreated with eugenol) (50 nm)	male Wistar rats	intraperitoneal injection of NP suspensions in the BSYS (1–10 mg/kg body weight)	hepatic and renal toxicity; toxicity attenuated by eugenol coadministration	(Wani et al. 2021)^143^
LNP (79–91 nm) Ch-LNP (129–219 nm)	adult wild-type tropical 5D zebrafish (Danio rerio)	XX injection/administration of NP suspensions in the BSYS. Dose: unspecified	Ch-LNP formulations exhibited greater toxicity than uncoated LNPs	(Stine et al. 2021)^144^
CuO NP (36.6 nm)	male and female Sprague–Dawley rats	oral gavage administration of NP suspensions to the BSYS (50 and 100 mg/kg)	dose- and sex-dependent toxicity: significant alterations in total bilirubin, liver enzymes (ALT, alkaline phosphatase), antioxidant enzymes, and total protein at both 50 and 100 mg/kg in male and female rats; histological damage to liver, heart, and lungs observed in males at the higher dose, while low-dose females were least affected	(Naz et al. 2021)^145^
ZnO NP (30–40 nm) CuO NP (25–55 nm) CeO_2_ NP (30–50 nm)	carrot (Daucus carota)	pots with BSYS watered once a week for 13 weeks with 0.05 L of NP suspension (1, 10, 100, and 1000 mg/L)	concentration-dependent root accumulation of metal-based nanostructures (1–1000 mg/L)	(Ebbs et al. 2016)^146^
CeO_2_ NP (25 nm) TiO2 NP (25 nm)	seedlings of Hordeum vulgare L. Caryopses	aqueous dispersion of NP (0, 500, 1000, and 2000 mg/L: 7 days)	genotoxicity, oxidative stress, and inhibition of root growth	(Mattiello et al. 2015)^147^
polystyrene NP (24 nm)	green algal lab. culture Zooplâncton (Daphnia magna) Crucian carp (Carassius carassius)	green algae were distributed in glass jars containing polystyrene nanoparticles at 0.01% (w/v)	trophic transfer associated with altered lipid metabolism in fish	(Cedervall et al. 2012)^108^

a BSYS: biological system; NP: nanoparticle; LNP: lipid nanoparticle; Ch-LNP: chitosan-coated lipid nanoparticle; WT: wild-type; Ogg1 KO: knockout of Ogg1; ICR: Institute of Cancer Research; HCC: human cancer cell line.

As reported in Table 4, this diversification has been accompanied not only by the investigation of a wider range of nanomaterials but also by the incorporation of increasingly complex biological models and toxicological end points. Beyond conventional mammalian systems, recent studies have expanded to include plants, aquatic organisms, and environmentally relevant exposure scenarios, reflecting growing concerns regarding the ecological risk and environmental sustainability. Similarly, nanotoxicological investigations have evolved beyond acute toxicity assessments to encompass biodistribution, organ-specific toxicity, genotoxicity, trophic transfer, and the effects of long-term exposure. Despite methodological heterogeneity across studies, physicochemical characteristics such as particle size, surface chemistry, and coatings, along with the exposure scenario and biological context, are the primary factors influencing the fate and toxicity of nanomaterials.^112,113,128^

### Proposed Framework for Nanotoxicity Assessment

The evidence discussed throughout this review highlights the need for an integrated approach to nanotoxicity assessment. On the basis of these advances and the remaining research gaps, we propose the conceptual framework shown in Figure 4. This framework should be interpreted as a conceptual strategy intended to guide nanotoxicological studies rather than as a single-assessment approach. First, characterization of the nanostructure is essential since properties such as particle size, shape, surface chemistry, aggregation state, and surface modifications influence biological interactions, biodistribution, and toxicological outcomes.^89,126,130^

**Figure 4:**
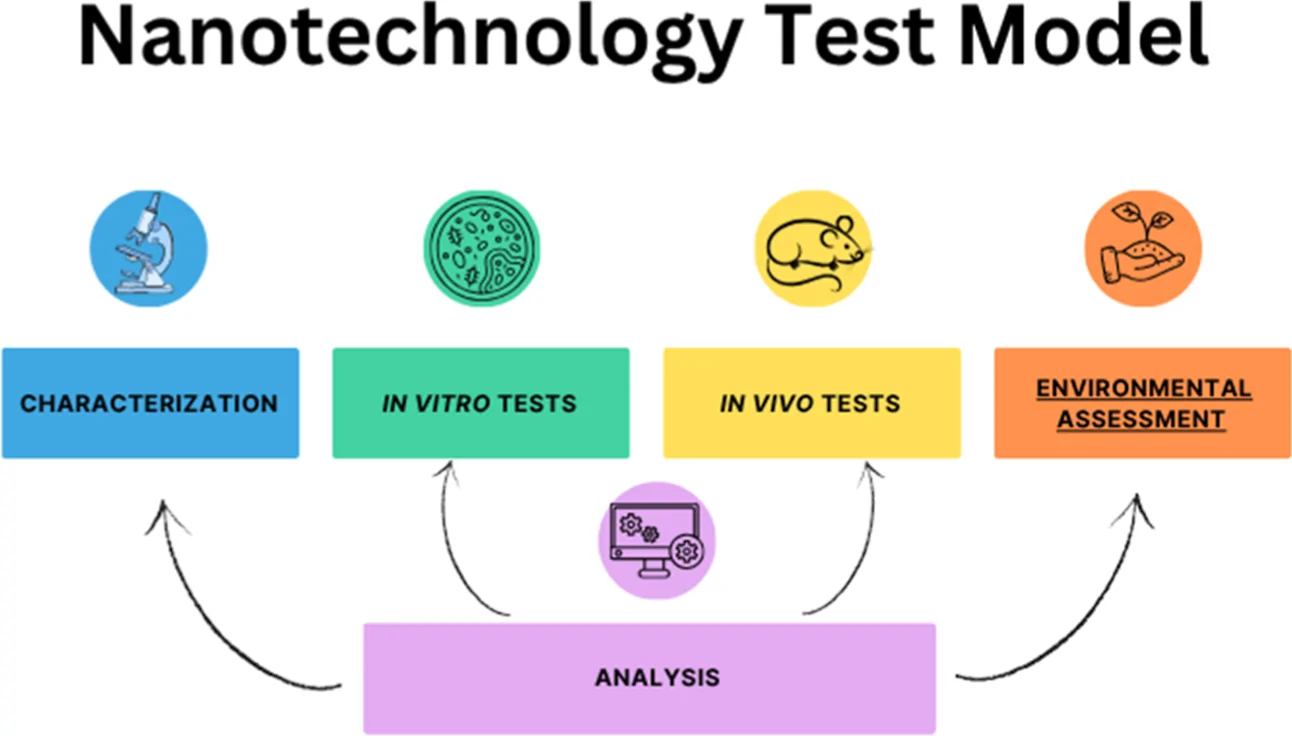
Proposal for a toxicological study of a nanostructure. Step 1—determination of size and distribution (DLS, electron microscopy), morphology (SEM, TEM), and surface charge (zeta potential); Step 2—3D cell culture models; Step 3—acute and chronic toxicity tests in rats following intravenous administration at the intended dosage; Step 4—environmental fate and biodegradation potential in soil or aquatic systems; and Step 5—integration of data using artificial intelligence and machine learning to correlate physicochemical properties with toxicity and construct predictive models.

Next, an in vitro study is a faster and lower-cost alternative that allows the use of fewer animals and the acquisition of preliminary results that can serve as a basis for future tests. Today, in vitro models using 3D cell cultures have shown promise compared to the classic 2D cell culture model. 3D cultures can better replicate cellular organization, tissue interactions, and gene expression, resulting in a greater physiological response.^115,116^ This model has reduced the gap between in vitro and in vivo studies, encouraging its application in nanotoxicology.

Regardless of the results from the 3D cellular model, in vivo studies remain essential for assessing biodistribution, toxicokinetics, biopersistence, systemic toxicity, and complex organism-level responses that current in vitro approaches cannot yet fully reproduce.^97,148^

To illustrate this stage of the framework, the rat model was selected for Figure 4 because rodents remain among the most widely used mammalian systems in toxicology and have generated extensive evidence relevant to nanosafety assessment.^23,29,40,59,60,110,117^ However, the proposed framework does not imply that a single animal model is appropriate for every type of nanosafety assessment. The intended application, route of exposure, toxicological outcome, and physiological relevance should guide the model selection. Mammalian models are important for evaluating biodistribution, toxicokinetics, organ-specific toxicity, and long-term effects, whereas alternative models, including zebrafish and *Caenorhabditis elegans*, may be more appropriate for specific mechanistic, developmental, or environmental end points. Furthermore, differences in anatomy, physiology, and health status can influence the distribution and toxicity of nanomaterials, emphasizing the importance of selecting experimental models that adequately reflect realistic exposure scenarios.^13,97,148^

The choice of administration route and dose should reflect scenarios of chronic exposure at low concentrations (e.g., in the μg L^–1^ range or less), as evidenced in the literature, rather than relying solely on acute high-dose protocols. Recent studies show that responses to nanoparticles at environmental or occupational levels, often in the parts per billion or parts per trillion range, are biologically relevant and require adaptation of the experimental design.^149^ Similarly, the duration of exposure must be determined based on the expected scenario, the nanomaterial’s persistence and environmental transformation, its biodistribution and clearance profile, the biological parameter under investigation, and the life stage of the exposed organism. Consequently, the literature does not provide a universal standard for chronic nanotoxicity studies; instead, it employs different experimental designs depending on the intended application. Repeated-dose studies have been used to simulate occupational exposure scenarios,^150^ whereas gestational exposure models have been adopted to investigate developmental toxicity following maternal transfer of TiO_2_ nanoparticles.^128^ Continuous exposure protocols in adult zebrafish have been used to assess long-term behavioral and biochemical changes,^151^ whereas life cycle and multigenerational designs in *Drosophila* have been employed to investigate cumulative and transgenerational effects.^152^ Test conditions that replicate biologically relevant exposure scenarios can reveal toxicological outcomes not observed in conventional acute studies, including progressive cellular accumulation, persistent oxidative stress, neurobehavioral alterations, developmental toxicity, and other sublethal effects.^153^

Finally, the assessment of the environmental fate must go beyond simplified laboratory tests conducted under ideal conditions with nanomaterials. Realistic assessments must consider environmentally relevant exposure concentrations, transformed rather than pristine nanomaterials, aggregation, dissolution, aging, protein corona formation, interactions with natural organic matter, chronic low-dose exposure, and trophic transfer through food chains, as these processes substantially modify the behavior, bioavailability, and toxicity of nanomaterials under real-world environmental conditions.^113^

Throughout the entire process, machine learning (ML) and artificial intelligence (AI) techniques can be used to correlate the properties of nanostructures with observed toxicity. ML algorithms can be used to predict critical physicochemical properties, such as size, surface charge, specific area, and chemical composition, from experimental data libraries and simulations. Deep learning and automated image processing techniques, used in parallel with in vitro assays, enable the extraction of morphological changes and cellular responses in a fast, reproducible, and scalable manner.^133^ In in vivo models, integrating ML with kinetic data, using models adapted from PBPK for particles or nano-QSTR, allows the estimation of internal doses, tissue distribution trajectories, and nonlinear or hormetic effects typical of nanoparticles.^133,154^ Furthermore, in the environmental and ecotoxicological context, machine-learning tools can predict the mobility, transformation, and persistence of nanomaterials in aquatic or terrestrial ecosystems.^120^ The iterative application of AI/ML can thus help to define more realistic, low-dose test scenarios. Rather than replacing experimental testing, these computational approaches should be viewed as complementary tools that enhance study design, support data interpretation, and improve the predictive nanosafety assessment.

Integrating physicochemical characterization, biologically relevant experimental models, realistic environmental assessment, and predictive computational approaches provides a more robust foundation for nanosafety assessment and supports the development of future regulatory decision-making frameworks. To ensure the reproducibility of studies, it is important to apply the test guidelines proposed by the OECD and, when available, the methodologies specified in the relevant ISO standards.

### Importance of Nanotoxicology in the Regulation of Nanotechnology

Despite ongoing research in nanotoxicology, global regulations for nanotechnology products have not yet been established. This regulatory failure presents a complex challenge, as there remains insufficient epidemiological evidence of the toxicity of nanomaterials, inconclusive toxicological data from experimental models, and limited information on the concentrations of these compounds in the air.^34^

The European Union and the United States are examples of nations with specific regulations that consider various sectors including health, food, and the environment. However, their regulatory agencies do not have well-established methods to control the features of nanomaterials, such as safety, physical and chemical characteristics, and quantities of nanostructures. Therefore, analyses are typically conducted for each of the cases.

In 2013, the global project NANoREG was established to regulate data collection to support managers in testing of manufactured nanomaterials. The European Union leads the project, which comprises laboratories from more than 85 European Union partners, as well as Australia, Canada, South Korea, the United States, Japan, and Brazil, among others. The team analyzed safety in the production and application of nanomaterials, focusing on product design, risk assessment, and regulatory preparedness.^155^ In parallel, several institutions work individually or collaboratively to provide guidelines and recommendations for the governance and oversight of nanotechnology, such as the Organization for Economic Cooperation and Development (OECD), the World Health Organization (WHO), the International Organization for Standardization (ISO), and the Center of Excellence in Nanotechnology Safety (SAFENANO).^34^

International initiatives by the ISO and the OECD have contributed to the standardization of methods for characterizing and toxicologically evaluating nanomaterials. ISO/TR 13014 of 2012 provides guidance on physicochemical characterization, an important factor in determining nanotoxicity.^156^ ISO 10993-22 provides guidelines for determining the biocompatibility of nanomaterials used in medical devices.^157^ The OECD series of test guidelines (TGs) includes nanomaterials for analyses of specific surface area (TG 124), particle size and distribution (TG 125), and dispersion stability (TG 318). The World Health Organization (WHO) recognizes and highlights the potential risks of micro- and nanoplastics to human and environmental health through its Plastics and Health Initiative and its active participation in negotiations for a global treaty on plastic pollution.^158^ The main international standards and guidelines currently used for nanomaterial characterization, testing, and risk assessment are summarized in Table 5.

**Table 5:** International Standards and Guidelines for the Regulation and Testing of Nanomaterials

organization	standard/guideline	title	purpose/scope	refs
ISO	ISO/TR 13014:2012	Nanotechnologies—Guidance on physicochemical characterization of engineered nanoscale materials for toxicologic assessment	provides guidance on physicochemical characterization to support toxicity assessment of nanomaterials	ISO (2024)^156^
ISO	ISO 10993-22:2017	Biological evaluation of medical devices—Part 22: Guidance on nanomaterials	provides principles for evaluating the biocompatibility of nanomaterials used in medical devices	ISO (2017)^157^
OECD	Test Guideline (TG) 124	Determination of the Specific Surface Area of Nanomaterials	establishes procedures for measuring specific surface area, a critical determinant of nanomaterial reactivity and toxicity	OECD (2022)^161^
OECD	Test Guideline (TG) 125	Determination of particle Size and Size Distribution of Nanomaterials	describes standardized methods to measure particle size and distribution of nanomaterials	OECD (2023)^162^
OECD	Test Guideline (TG) 318	Stability assessment of Nanomaterial dispersions in Simulated Environmental Media	evaluates the dispersion stability of nanomaterials under environmentally relevant conditions	OECD 2017 ^163^
WHO	Plastics and Health Initiative (2022)	Global framework addressing health and environmental risks of micro- and nanoplastics	promotes international coordination and policy development to mitigate the impact of nano- and microplastics on health	WHO (2023)^158^

More recently, regulatory discussions have expanded to risk assessment, encompassing the concepts of safety and sustainability by design (SSbD) and the OECD’s safe and sustainable innovation approach (SSIA). These frameworks promote integrating safety, environmental sustainability, and regulatory considerations from the early stages of material development, rather than waiting until potential risks are identified, to favor the development of technologically effective nanomaterials that are safer for human and environmental health.^31^

To illustrate, the Nanotechnology Advisory Committee (CCNano) and the Interministerial Committee for Nanotechnology (CIN) from Brazil contribute to national discussions on nanotechnology policy and governance. Currently, bills are being processed to establish a National Nanotechnology Policy and to require the labeling of products that use nanotechnology. Resolution of the Collegiate Board of Directors—RDC N° 326, on December 3, 2019, from the Brazilian Health Regulatory Agency (ANVISA) addresses additives intended for manufacturing plastic materials and polymeric coatings in contact with food. The standard establishes the nanostructures authorized for use in coatings that come into contact with food, along with their migration limits.^159^

However, there are still no globally harmonized and legally binding safe exposure limits for nanomaterials. The described regulatory fragmentation is clarified by the case of titanium dioxide (TiO_2_) as a food additive (E171). In 2021, the European Food Safety Authority (EFSA), considering the particulate nature of E171 and employing a risk assessment specific to nanomaterials, concluded that the additive could no longer be considered safe because of evidence of genotoxicity, accumulation potential, and immunotoxicity (EFSA FAF Panel, 2021). This assumption prompted the European Commission to ban the use of TiO_2_ as a food additive in the EU. In contrast, other regulatory agencies, such as the U.S. Food and Drug Administration (FDA), Health Canada, and the UK Food Standards Agency, do not consider safety concerns regarding food-grade TiO_2_ because their assessment frameworks do not incorporate specific nanoscale considerations.^160^ This divergence, in which a single compound is classified as unsafe by one regulatory body and safe by several others based on distinct methodological frameworks rather than new experimental evidence, exemplifies why harmonized regulatory criteria specific to nanomaterials remain unresolved challenges in the field.

This fragmentation, together with the limited availability of robust long-term data, further complicates regulatory decision making and the establishment of risk control parameters. One of the main obstacles is the intrinsic complexity of these materials. Unlike conventional chemicals, they do not constitute a single regulatory class. Even nanomaterials with identical chemical compositions may exhibit different toxicological profiles, depending on their physicochemical characteristics, making it scientifically difficult to determine universal exposure thresholds. Consequently, their toxicity cannot be predicted solely by their chemical composition since biological responses are strongly influenced by particle size, shape, surface chemistry, agglomeration state, protein corona formation, environmental transformations, and exposure pathway.

Furthermore, the nanotoxicology literature is still characterized by heterogeneity in experimental models, characterization methods, exposure conditions, and evaluated end points, which limits direct comparisons between studies and hinders the establishment of widely applicable risk criteria. As a result, regulatory agencies often adopt case-by-case assessments, rather than generic exposure standards. Consequently, although regulatory harmonization has advanced substantially in the development of characterization methods and testing guidelines, the lack of sufficiently robust, comparable scientific evidence continues to prevent the establishment of universally applicable regulatory limits.

Addressing these challenges will require a regulatory framework grounded in several key points, including the characterization of nanostructures; the use of reproducible, precise methodologies; the assessment and management of risks associated with nanomaterials; and collaboration among researchers, industry, and regulatory agencies as well as international coordination of efforts for this purpose. Future regulatory frameworks will require integrating the technical advances already consolidated by the ISO and the OECD, research findings from databases, and global public health strategies while addressing human, environmental, and occupational health concerns.

## Final Considerations

Over the past two decades, significant progress has been made in nanotoxicology driven by the development of increasingly sophisticated experimental, computational, and regulatory approaches for toxicological assessment. Various nanostructures have been assessed under diverse conditions by using different organisms and ecological and biological models.

Despite significant advancements in nanotechnology, the complex interactions between nanostructures and biological or environmental systems present challenges that must not be overlooked. Our understanding of the dynamic interactions between nanomaterials and biological or environmental systems remains incomplete, highlighting the need for standardized testing protocols to ensure a consistent and reproducible environment. While many nanostructures clearly exhibit toxicity under specific conditions, determining safe concentration thresholds and establishing consistent exposure scenarios are crucial issues that demand our attention and resolution.

Owing to the multifaceted nature of nanotoxicity, artificial intelligence and machine learning tools have increasingly been utilized to support data integration and risk assessment. On the basis of the reviewed literature, several key insights have emerged: (i) particle size significantly influences toxicity, and smaller nanostructures generally exhibit greater reactivity and biological impact; (ii) biodegradable nanostructures are typically more easily recognized and tolerated by biological systems; (iii) functionalization with biocompatible compounds, such as specific polymers, can help reduce adverse effects; (iv) protein corona formation dynamically alters the biological identity of nanomaterials, thereby influencing their biodistribution, cellular interactions, and toxicity; (v) nanostructures can bioaccumulate in plants and aquatic organisms, facilitating trophic transfer; and (vi) environmental transformations and trophic transfer substantially influence nanomaterial persistence, bioavailability, and ecological risk.

It is necessary to use physiologically relevant biological systems that reproduce the dynamic processes of protein corona formation, dissolution, aggregation, and biotransformation. Furthermore, realistic doses of environmental or consumer exposure should be applied and chronic exposure should be considered. The nanostructure under study must be consistently characterized, not only in the initial testing phase but throughout the entire process and post-testing. The application of machine learning tools, supported by high-quality, standardized data sets and environmentally relevant conditions, is essential for understanding the dynamic behavior of nanomaterials, conducting reliable toxicity assessments, and establishing standardized testing protocols that ensure safety and efficacy across all applications.

This review presents a synthesis of 20 years of nanotoxicology research, highlighting both our progress and areas that still require attention. While we have gained considerable insights, there remains an opportunity to strengthen our understanding further and support the development of global regulatory frameworks and safe and sustainable innovation strategies. Given the increasing production and use of these materials across various industries, it is vital to establish comprehensive toxicity criteria and safe exposure limits that account for their entire life cycle. By fostering collaboration among academia, industry, and regulatory agencies, we can work together to achieve an important goal. The scientific community is increasingly equipped with the knowledge, experimental methodologies, and computational tools needed to advance nanotoxicology, fostering the development of safer nanomaterials, more reliable risk-assessment strategies, and internationally harmonized regulatory frameworks.
